# Bleeding and thrombosis

**DOI:** 10.1093/ehjcvp/pvaf017

**Published:** 2025-05-02

**Authors:** Stefan Agewall

**Affiliations:** Institute of Clinical Sciences, Karolinska Institute of Danderyd, Stockholm, Sweden

FXI inhibition is highly topical. In this issue of the journal, we start with an invited editorial by Dr Gragnano *et al.* commenting on the AZALEA—TIMI 71 trial^1^ and the OCEANIC—AF trial.^2^^,^^3^ In another invited editorial, Galli *et al.* discuss the effects of early administration of colchicine in patients with STEMI.^4^^,^^5^

Multiple anti-inflammatory drugs have been tested for secondary prevention after myocardial infarction (MI), giving mixed results and questioning the efficacy of anti-inflammatory therapy.^6^^,^^7^ No head-to-head comparisons between anti-inflammatory drugs have been performed. In a study by Dr Capodanno *et al.*, they aimed to compare the efficacy and safety of anti-inflammatory drugs for secondary prevention after MI and the relative merits of specific drugs and administration strategies. Twenty-eight randomized studies, involving 44 406 patients with a mean follow-up of 11 months, were included. The authors concluded that in secondary prevention of MI, anti-inflammatory therapy significantly reduces MACE without increasing serious adverse events, but it is associated with an increased risk of gastrointestinal adverse events.

There are different antiplatelet strategies in carriers of CYP2C19 loss-of-function alleles.^8^ The POPular Genetics trial showed in a randomized setting that a CYP2C19 genotype-guided de-escalation strategy reduced the risk of bleeding without affecting ischaemic risk, compared to standard DAPT in patients with ST-elevation myocardial infarction (STEMI).^9^ van den Broek and co-workers from the Netherlands estimated the cost-effectiveness of this personalized approach compared to standard dual antiplatelet therapy (DAPT; aspirin plus ticagrelor/prasugrel). The authors concluded that a genotype-guided de-escalation strategy led to an increase of 55.30 quality-adjusted life years and saved €698 286 compared to standard DAPT based on a lifetime horizon.

Amiodarone is a moderate inhibitor of cytochrome P450 3A4 (CYP3A4) and P-glycoprotein (P-gp) enzymes.^10^^,^^11^ Accordingly, pharmacokinetic studies have shown increased direct oral anticoagulants (DOAC) drug levels with concurrent amiodarone.^12^ Dr Shurrab and co-workers from Canada conducted a meta-analysis to compare bleeding risk among patients receiving DOACs, with and without concurrent amiodarone. Nine studies were identified, which included 124 813 patients on amiodarone/DOACs, and 314 074 on DOACs. The conclusion of the study was that concurrent use of amiodarone and DOACs was associated with a modest increase in major bleeding.

Transcatheter mitral valve replacement has become a feasible alternative to surgical mitral valve replacement in selected patients at high surgical risk.^13^ Dr Cahill *et al.* from the UK found 47 studies (6170 patients, total follow-up 9541.8 patient-years). The overall incidence of bioprosthetic mitral valve thrombosis (bMVT) was 5.0% per 100-patient-years. The study group concluded that bMVT is not uncommon, with a numerically higher incidence in transcatheter compared to surgical valves, but the comparison was not statistically significant. VKAs are associated with a lower incidence of bMVT compared to DOACs.

Approximately 30–50% of treated hypertensive patients fail to reach target BP levels,^14^ highlighting the need for novel therapeutic strategies. Dr Ngoc Le *et al.* from the UK aimed to explore the effects of phosphodiesterase type 5 inhibitors,^15^ soluble guanylate cyclase stimulators, endothelin receptor antagonists,^16^ and angiotensinogen inhibitors on a range of CKM outcomes. Mendelian randomization (MR), summary-based MR, and colocalization analyses were applied to assess the drug effect on coronary artery disease, MI, ischaemic stroke, atrial fibrillation, heart failure, type 2 diabetes (T2D), and chronic kidney disease. Genetic association and gene expression summary data were obtained from the largest European-ancestry genome-wide association studies and the Genotype-Tissue Expression version 8 for 29 tissues relevant to the outcomes' pathophysiology. Genetically predicted systolic blood pressure reduction was associated with reduced risks of all outcomes.

Cardiologists have only had rare exposure to haemophilia patients and patients with other congenital bleeding disorders during the last decades, as these patients had a reduced life expectancy and were partly protected against thrombosis due to the bleeding disorder. In a review paper, Dr Atar and co-workers aimed at providing clinical consensus statements on optimizing antithrombotic treatment strategies for patients with congenital bleeding disorders and highlighting the current gaps in knowledge in these complex clinical settings.

The never-ending need for more effective and safer pharmacological strategies continues. In a review by Dr Tamargo and co-workers, the most relevant studies in cardiovascular pharmacotherapy in 2024 are summarized, including the approval of first-in-class drugs for the treatment of resistant hypertension and pulmonary arterial hypertension, label expansions for bempedoic acid and semaglutide, and the results of major randomised clinical trials (RCTs) that have met the prespecified primary endpoints, thereby filling some gaps in knowledge and opening new perspectives in the management of cardiovascular disease (CVD), and those RCTs whose results did not confirm the proposed research hypotheses. The authors also include a section on drug safety, where the newest data on adverse reactions and drug-drug interactions are presented. Finally, the most important ongoing phase 2 and phase 3 clinical trials assess the efficacy and safety of cardiovascular drugs for the prevention and treatment of CVD.
